# YAP promotes erlotinib resistance in human non-small cell lung cancer cells

**DOI:** 10.18632/oncotarget.10458

**Published:** 2016-07-07

**Authors:** Ping-Chih Hsu, Bin You, Yi-Lin Yang, Wen-Qian Zhang, Yu-Cheng Wang, Zhidong Xu, Yuyuan Dai, Shu Liu, Cheng-Ta Yang, Hui Li, Bin Hu, David M. Jablons, Liang You

**Affiliations:** ^1^ Thoracic Oncology Laboratory, Department of Surgery, Comprehensive Cancer Center, University of California, San Francisco, CA, USA; ^2^ Department of Thoracic Medicine, Chang Gung Memorial Hospital, Linkou, Taoyuan, Taiwan; ^3^ Department of Thoracic Surgery, Beijing Chao-Yang Hospital, Affiliated with Capital University of Medical Science, Beijing, People's Republic of China

**Keywords:** Hippo pathway, yes-associated protein, epidermal growth factor receptor tyrosine kinase inhibitor (EGFR-TKI) resistance, erlotinib, non-small cell lung cancer

## Abstract

Yes-associated protein (YAP) is a main mediator of the Hippo pathway, which promotes cancer development. Here we show that YAP promotes resistance to erlotinib in human non-small cell lung cancer (NSCLC) cells. We found that forced YAP overexpression through YAP plasmid transfection promotes erlotinib resistance in HCC827 (exon 19 deletion) cells. In YAP plasmid-transfected HCC827 cells, GTIIC reporter activity and Hippo downstream gene expression of AREG and CTGF increased significantly (P<0.05), as did ERBB3 mRNA expression (P<0.05). GTIIC reporter activity, ERBB3 protein and mRNA expression all increased in HCC827 erlotinib-resistance (ER) cells compared to parental HCC827 cells. Inhibition of YAP by small interfering RNA (siRNA) increased the cytotoxicity of erlotinib to H1975 (L858R+T790M) cells. In YAP siRNA-transfected H1975 cells, GTIIC reporter activity and downstream gene expression of AREG and CTGF decreased significantly (P<0.05). Verteporfin, YAP inhibitor had an effect similar to that of YAP siRNA; it increased sensitivity of H1975 cells to erlotinib and in combination with erlotinib, synergistically reduced migration, invasion and tumor sphere formation abilities in H1975 cells. Our results indicate that YAP promotes erlotinib resistance in the erlotinib-sensitive NSCLC cell line HCC827. Inhibition of YAP by siRNA increases sensitivity of erlotinib-resistant NSCLC cell line H1975 to erlotinib.

## INTRODUCTION

Epidermal growth factor receptor (EGFR) gene mutations are detected in 10% to 30% of patients with non-small cell lung cancer (NSCLC) [1]. In clinical trials, the EGFR tyrosine kinase inhibitor (EGFR-TKI) erlotinib has shown a higher response rate, longer progression-free survival and lower toxicity than conventional chemotherapy [2, 3]. Therefore, erlotinib has been used as a first-line treatment for advanced lung adenocarcinoma harboring sensitive EGFR mutations such as exon 19 deletion and L858R. However, the vast majority of NSCLC tumors become resistant to EGFR-TKI treatment because of the occurrence of resistant mutations such as T790M in EGFR [4, 5].

The Hippo (also known as the Salvador-Warts-Hippo) pathway, a known cancer pathway, was recently identified in NSCLC [6, 7]. An important mediator protein in the Hippo pathway is Yes-associated protein (YAP), which promotes cancer development [8–10], and has been suggested as a potential drug target for melanoma, mesothelioma and hepatocellular carcinoma [11–14]. K-ras, mitogen-activated protein (MAP)-ERK kinase (MEK), and Extracellular signal-regulated kinase (ERK) signaling are downstream signaling of EGFR [15–18], and we recently reported crosstalk between Hippo/YAP and EGFR/ERK signaling pathways in human NSCLC cells [19]. In 2007, Engelman et al. reported that activation of ERBB3 is one mechanism of resistance in gefitinib-resistant cells, which were derived from the NSCLC cell line HCC827 (exon 19 deletion) [20]. Recently, He at al. reported that YAP induces the expression of epidermal growth factor (EGF) receptors including EGFR and ERBB3 in ovarian cell lines [21,22].

In this study, we sought to investigate whether YAP promotes erlotinib resistance in human NSCLC and whether the ERBB3 expression increased after YAP up-regulation.

## RESULTS

### Forced overexpression of YAP promotes resistance to erlotinib in HCC827 cells

**Figure 1:**
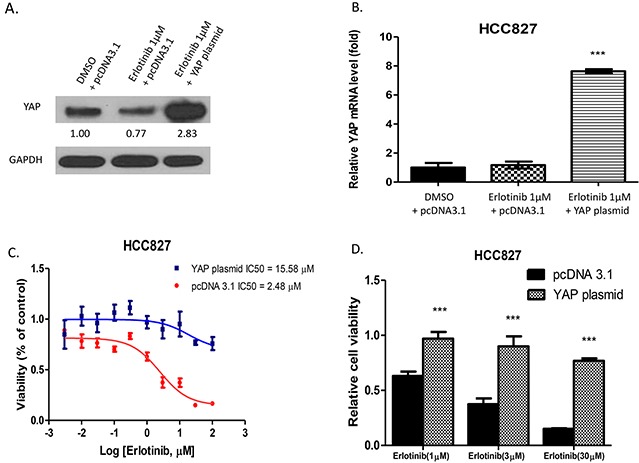
Forced overexpression of YAP in HCC827 promotes resistance to erlotinib in HCC827 cells **A.** Western blotting showed that YAP protein expression increased in YAP plasmid-transfected HCC827 after erlotinib treatment. **B.** YAP mRNA expression increased more in HCC827 cells with YAP forced overexpression than in pcDNA 3.1 transfected HCC827 cells after erlotinib treatment and DMSO control treatment (***P < 0.001). **C.** The IC50 of erlotinib was 15.58μM for cells transfected with YAP plasmid, and 2.48 μM for cells transfected with pcDNA 3.1. **D.** After treatment with erlotinib, cell viability of YAP plasmid-transfected HCC827 cells increased compared to pcDNA3.1-transfected HCC827 cells (***P < 0.001).

To investigate whether YAP promotes resistance to erlotinib in HCC827 cells, we forced YAP overexpression by transfecting YAP plasmid in HCC827 cells. The cells transfected with pcDNA 3.1 were used as the control. Western blotting showed that after 24-hour erlotnib treatment, YAP protein level decreased in pcDNA 3.1-transfected HCC827 cells, and increased in YAP plasmid-transfected HCC827 cells (Figure 1A). Analysis of YAP mRNA level with real-time PCR showed that after 24-hour erlotinib treatment in YAP plasmid-transfected HCC827 cells, the YAP mRNA expression level increased over 7 times more than after erlotinib treatment in pcDNA 3.1-transfected HCC827 cells and DMSO-control cells (P<0.001) (Figure 1B). The transfected cells were then treated with erlotinib at a titrated concentration for cell viability assay. The IC50 of erlotinib was 2.48 μM for HCC827 cells transfected with pcDNA 3.1 and 15.58μM for for HCC827 cells transfected with YAP plasmid (Figure 1C). The cell viability of pcDNA 3.1 transfected cells decreased significantly by 33%, 52% and 61% at 1μM, 3μM, and 30μM of erlotinib, respectively, compared to HCC827 cells transfected with YAP plasmid (P<0.001) (Figure 1D).

### YAP protein expression increased in erlotinib-resistant HCC827 cells

To investigate whether YAP protein expression increases in erlotinib-resistant HCC827 cells, we generated HCC827 erlotinib resistant (ER) cells. Western blotting showed that YAP protein expression increased in these cells when compared to parental HCC827 cells (Supplementary Figure S1A). After erlotinib treatment, YAP protein decreased dramatically in parental HCC827 cells, but increased in HCC827 ER cells (Supplementary Figure S1C). The p-YAP/YAP ratio increased significantly in parental HCC827 cells after 1.0 and 10.0 μM erlotinib treatment (P < 0.001), but did not change in HCC827 ER cells (Supplementary Figure S1D). Moreover, merlin (NF2), LATS1 protein expression and p-YAP/YAP ratio decreased in HCC827 ER cells compared to parental HCC827 cells (Supplementary Figure S1A, S1B).

### Inhibition of YAP by SiRNA enhanced the cytotoxicity of erlotinib to H1975 cells

**Figure 2:**
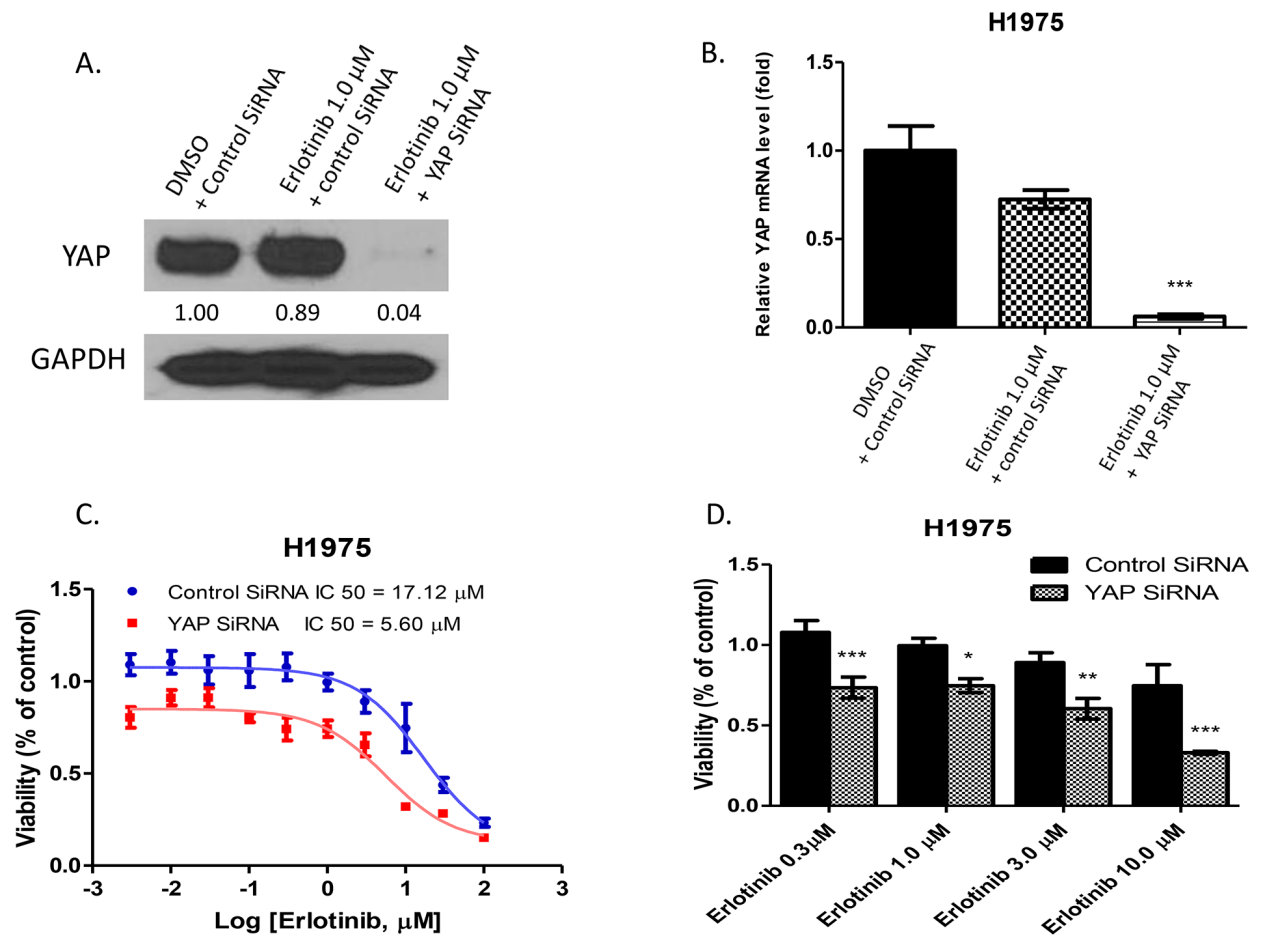
Inhibition of YAP by siRNA enhanced the cytotoxicity of erlotinib to H1975 cells **A.** Western blotting showed that YAP protein expression level decreased after YAP siRNA transfection and erlotinib treatment in H1975 cells. **B.** YAP mRNA expression significantly decreased in H1975 cells with YAP siRNA transfection after erlotinib treatment (***P < 0.001). **C.** The IC50 of erlotinib was 5.60μM for H1975 with YAP silencing by YAP siRNA, and 17.12μM for H1975 transfected by control siRNA. **D.** After treatment with erlotinib, cell viability of H1975 cells with YAP silencing by YAP siRNA decreased compared to control siRNA-transfected H1975 cells (*P < 0.05, **P < 0.01, ***P < 0.001).

To investigate whether YAP inhibition enhances the cytotoxicity of erlotinib in the NSCLC cell line H1975, these cells were treated with small interfering RNA (siRNA) to knock down YAP expression. YAP protein expression assayed by western blotting showed the protein level decreased after YAP knockdown (Figure 2A). Analysis of YAP mRNA expression with real-time PCR showed that in H1975 cells with YAP siRNA transfection, YAP mRNA decreased significantly after 1.0μM of erlotinib when compared to H1975 cells with control siRNA transfection after either DMSO or 1.0μM of erlotinib (P<0.001) (Figure 2B). After siRNA transfection, H1975 cells were treated with erlotinib at titrated concentrations for cell viability assay. The IC50 of erlotinib was 5.60 μM for YAP siRNA-transfected H1975 cells, and 17.12μM for control siRNA-transfected H1975 cells (Figure 2C). The cell viability of H1975 cells after YAP knockdown decreased significantly by 34%, 25%, 29%, and 41% at 0.3μM, 1.0μM, 3.0μM and 10.0μM of erlotinib, as compared to H1975 cells transfected with control siRNA (Figure 2D).

### The YAP inhibitor verteporfin increases the sensitivity of H1975 cells to erlotinib

**Figure 3:**
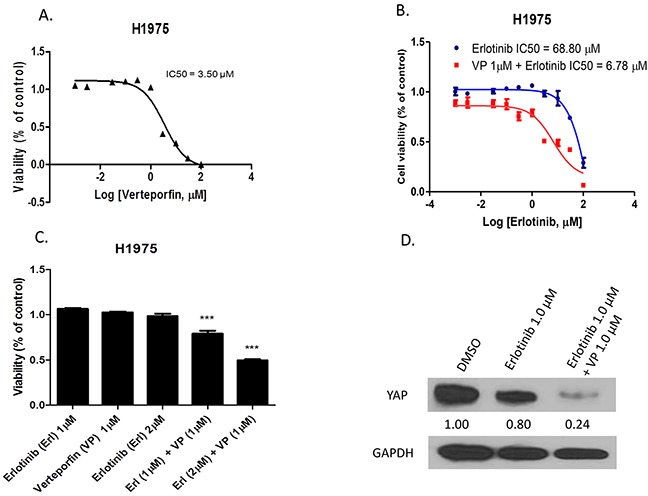
The YAP inhibitor verteporfin increased sensitivity of H1975 cells to erlotinib **A.** Cell viability analysis in H1975 cells after verteporfin treatment. **B.** Cell viability analysis of H1975 cells after erlotinib combined with 1μM verteporfin versus erlotinib alone. **C.** Cell viability of H1975 cells significantly decreased after combination treatment (***P < 0.001). **D.** Western blotting shows YAP protein expression level decreased after combination treatment in H1975 cells.

To investigate whether the YAP inhibitor verteporfin has an effect similar to that of YAP siRNA in increasing sensitivity of H1975 cells to erlotinib, we added verteporfin treatment in a viability assay of H1975 cells treated with erlotinib. When the cell viability of H1975 cells treated by verteporfin alone was assayed, the IC50 of verteporfin was 3.50μM in H1975 cells (Figure 3A). The IC50 of erlotinib was 68.80μM in H1975 cells, and decreased to 6.78μM when erlotinib was combined with 1μM verteporfin (Figure 3B). The combination of 1μM erlotinib and 1μM verteporfin decreased viability 19% - 27% more than 1μM erlotinib, 1μM verteporfin or 2μM erlotinib alone (P<0.001). The combination of 2μM erlotinib and 1μM verteporfin decreased viability by 49% - 56% more than 1μM erlotinib, 1μM verteporfin or 2μM erlotinib alone (P<0.001) (Figure 3C). Western blot analysis of YAP protein expression indicated that protein level decreased after the combined treatment in H1975 cells compared to DMSO control or erlotinib alone (Figure 3D).

### GTIIC reporter activity and Hippo pathway downstream gene expression in HCC827 and H1975 cells

**Figure 4:**
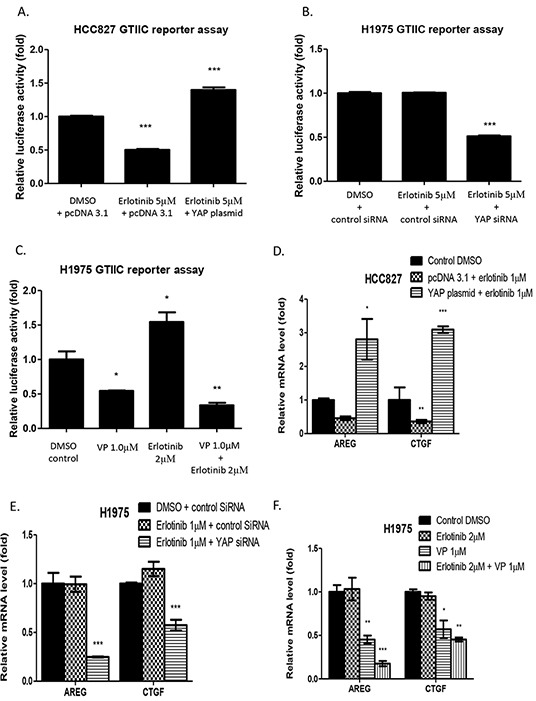
Hippo reporter activity and downstream gene expression in HCC827 and H1975 cells **A.** GTIIC reporter activity of the hippo pathway significantly decreased in HCC827 cells after erlotinib treatment and increased with YAP forced overexpression (***P < 0.001). **B.** GTIIC reporter activity significantly decreased in YAP siRNA-transfected H1975 cells after erlotinib treatment (***P < 0.001). **C.** GTIIC reporter activity significantly decreased in H1975 cells after verteporfin alone and after combined treatment with verteporfin and erlotinib (*P < 0.05, **P < 0.01). **D.** Hippo downstream gene expression of AREG and CTGF in HCC827 cells with YAP forced overexpression significantly increased even after erlotinib treatment (*P < 0.05, **P < 0.01, ***P < 0.001). **E.** Hippo downstream gene expression of AREG and CTGF in YAP siRNA-transfected H1975 cells significantly decreased after erlotinib treatment (***P < 0.001). **F.** Hippo downstream gene expression of AREG and CTGF significantly decreased after verteporfin treatment alone and after combined treatment with erlotinib and verteporfin treatment (*P < 0.05, **P < 0.01, ***P < 0.001).

The GTIIC reporter assay and real-time PCR for downstream gene mRNA analysis were used to verify whether GTIIC Hippo reporter activity and Hippo pathway downstream gene expression increase in HCC827 cells with YAP forced overexpression, and decrease in H1975 cells after YAP inhibition. In HCC827 cells with control plasmid, GTIIC reporter activity decreased after 24-hour treatment with 5μM erlotinib compared to DMSO treatment. GTIIC reporter activity increased when YAP was overexpressed after 5μM erlotinib treatment (Figure 4A). In H1975 cells, GTIIC reporter activity significantly decreased by 48% after the combination of YAP siRNA and 5μM erlotinib treatment compared to either DMSO control or 5μM erlotinib in control siRNA transfected H1975 cells (Figure 4B). GTIIC reporter activity significantly decreased by 45% after 1μM verteporfin alone and by 67% after 1μM vertiporfin and 2μM erlotinib combined as compared to DMSO control. GTIIC reporter activity increased after 2μM erlotnib alone (Figure 4C).

Analysis of the Hippo/YAP downstream genes AREG and CTGF by real-time PCR showed that in HCC827cells, AREG and CTGF mRNA expression level decreased after erlotinib treatment, and increased under YAP forced overexpression (Figure 4D). AREG and CTGF mRNA levels significantly decreased in YAP siRNA transfected H1975 cells after 1μM erlotinib treatment compared to control siRNA transfected H1975 cells treated with DMSO or 1μM erlotinib (Figure 4E). In H1975 cells, AREG and CTGF mRNA levels significantly decreased after verteporfin alone and after vertiporfin combined with erlotinib compared to erlotinib alone (Figure 4F).

GTIIC reporter activity significantly increased by about 3-fold in HCC827 ER cells when compared to parental HCC827 cells (Supplementary Figure S2B).

### Combination treatment with verteporfin and erlotinib restrains migration, invasion, and sphere formation of H1975 cells

**Figure 5:**
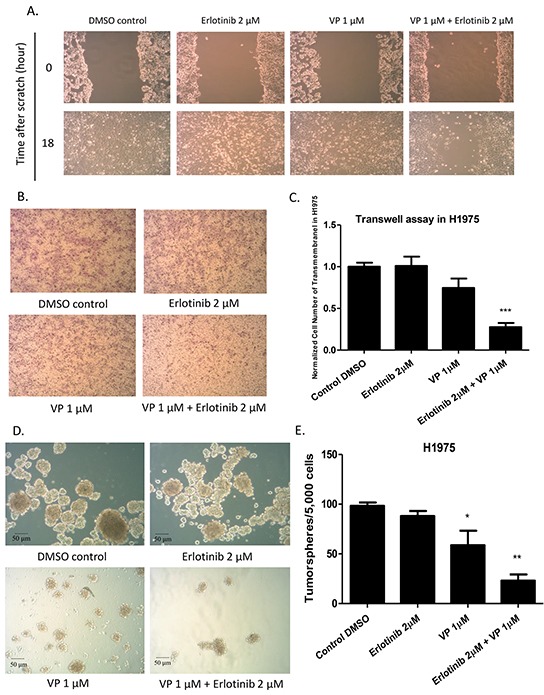
Analysis of cell migration, invasion and tumor sphere formation abilities after erlotinib alone, verteporfin alone and combination treatment with verteporfin and erlotinib in H1975 cells **(A)** Decrease in cell migration ability in H1975 cells after combination treatment **(B)** Decrease in cell invasion ability in H1975 cells after combination treatment **(C)** Quantitative analysis of transwell invasion assay result, indicating combination treatment decreased cell invasion ability in H1975 cells (***P < 0.001). **(D)** Decrease in tumor sphere formation ability in H1975 cells after verteporfin treatment alone, and after combined treatment with verteporfin and erlotinib. **(E)** Quantitative analysis shows verteporfin treatment alone or combination treatment decreased tumorsphere formation ability in H1975 cells (*P < 0.05, **P < 0.01).

Having verified the effect of verteporfin in decreasing GTIIC reporter activity and Hippo downstream gene expression, and in increasing sensitivity of H1975 cells to erlotinib, we further tested the efficacy of combined treatment with verteporfin and erlotinib in restraining migration, invasion, and sphere formation abilities in H1975 cells. To test the effect of this combination on the migration ability of H1975 cells, the cells were scratched with a 200 μl pipette tip, and then treated with DMSO, 2μM erlotinib alone, 1μM verteporfin alone, or a combination of 1μM verteporfin and 2μM erlotinib. Wound closure was observed after 18 hours of treatment, when the cells in the DMSO, 2μM erlotinib treatment alone, and 1μM verteporfin treatment alone groups were proximally confluent. Migration ability decreased in the cells treated with the combination of erlotinib and verteporfin (Figure 5A). To assess the effect of combined treatment on the invasion ability of H1975 cells, a transwell assay was performed after 2μM erlotinib, 1μM verteporfin, and the two treatments combined. The number of the cells that invaded the lower side of the membrane decreased significantly 24 hours after combination treatment when compared to that in the groups treated with DMSO control, 1μM verteporfin, or 2μM erlotinib (Figure 5B, 5C; P < 0.001). Next, we used a tumorsphere assay to measure the self-renewal of cancer stem cells in H1975 cells. H1975 tumorspheres were treated with DMSO, 2μM erlotinib alone, 1μM verteporfin alone, or a combination of 1μM verteporfin and 2μM erlotinib. Tumorsphere forming efficiency was significantly decreased by 1μM verteporfin alone and by combined treatment. (Figure 5D, 5E).

### YAP protein expression increased after time-dependent erlotinib treatment in HCC827 and H1975 cells, and ERBB3 expression increased with up-regulation of YAP in HCC827 cells

**Figure 6:**
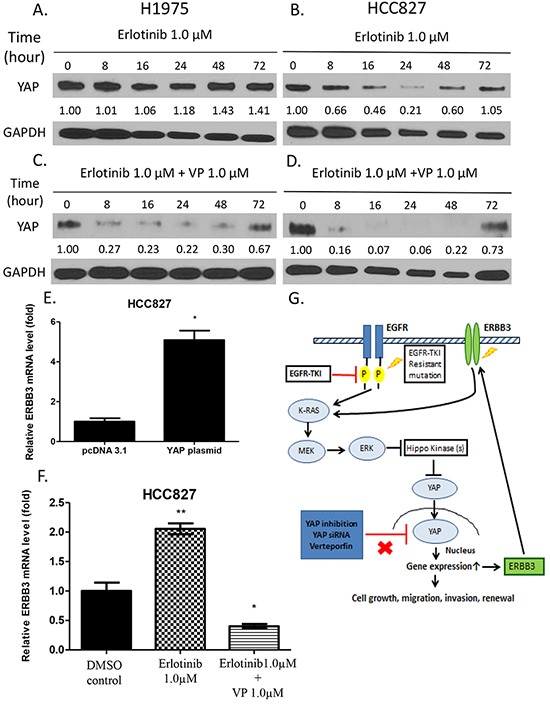
Western blot analysis of YAP protein expression after time-dependent treatment with erlotinib alone and combined treatment with verteporfin and erlotinib ERBB3 mRNA expression after YAP forced overexpression, erlotinib treatment and combination treatment with verteporfin and erlotinib. **A.** YAP protein level in H1975 cells increased after 48 and 72 hours of erlotinib treatment. **B.** In HCC827 cells, YAP protein level decreased after 8 to 24 hours of erlotinib treatment and rebounded after 48 and 72 hours of treatment. **C.** YAP protein level decreased in H1975 cells after combined treatment for 8, 16, 24 and 48 hours and rebounded after 72 hours. **D.** In HCC827 cells, YAP protein level decreased after combined treatment for 8, 16, 24, and 48 hours and then rebounded after 72 hours. **E.** ERBB3 mRNA expression significantly increased in HCC827 cells with YAP forced overexpression (*P < 0.05). **F.** ERBB3 mRNA expression significantly increased after erlotinib 1μM treatment and decreased after combination treatment (**P < 0.01,*P < 0.05,). **G.** A schematic diagram of a hypothetical model of how YAP promotes erlotinib resistance. In HCC827 cells, activation of YAP forms an autocrine loop with the ERBB3 pathway to bypass the EGFR signaling pathway after continuous erlotinib treatment. In H1975 cells, inhibition of YAP by siRNA or the YAP inhibitor verteporfin enhances the sensitivity to erlotinib.

To further investigate variation in YAP protein expression after erlotinib treatment at different time points in H1975 and HCC827 cells, we harvested the cells at 0, 8, 16, 24, 48 and 72 hours after erlotinib treatment. In H1975 cells, the YAP protein level increased at the 48-hour and 72-hour time point. In HCC827 cells, the YAP protein level decreased in a sustained manner after 8 to 24 hours of treatment, but rebounded after 48 hours of treatment (Figure 6A, 6B). In HCC827 and H1975 cells harvested at 0, 8, 16, 24, 48 and 72 hours after combined treatment with erlotinib and veteporfin, the YAP protein level decreased at 8, 16, 24, 48 hours, and then slightly increased at 72 hours. (Figure 6C, 6D). These results indicated that in both cell lines, YAP protein rebounded after continuous exposure to erlotinib alone, and that erlotinib and verteporfin combined resulted in a greater YAP degradation than erlotinib alone.

To study the potential outcome of YAP upregulation after prolonged treatment in HCC827 cells, transcriptional expression of ERBB3 was analyzed in HCC827 cells using real-time PCR. When YAP was overexpressed in the cells, ERBB3 mRNA expression level was significantly increased by 8-fold compared to that in the control cells (P<0.01) (Figure 6E). ERBB3 mRNA expression level increased after erlotinib treatment alone, and decreased after combined treatment with erlotinib and verteporfin (Figure 6F). In addition, in HCC827 ER cells, ERBB3 expression increased at the protein and mRNA level when compared to parental HCC827 cells. In HCC827 ER cells ERBB3 increased after dose-dependent erlotinib treatment (Supplementary Figure S1A, S1C, S2C).

## DISCUSSION

Our study provides several lines of evidence to support that YAP promotes EGFR-TKI erlotinib resistance in NSCLC. We found that forced overexpression of YAP in HCC827 cells promotes resistance to erlotnib, and that inhibition of YAP by YAP siRNA increases the cytotoxicity of erlotinib to H1975 cells. In addition, we found that YAP protein expression increased in HCC827 ER cells. Moreover, ERBB3 protein and mRNA expression also increased in HCC827 ER cells compared to parental HCC827 cells.

NSCLC tumors harboring exon 19 deletion become resistant to erlotinib because acquired resistance usually develops. For instance, T790M, known as a “gate keeper” mutation in the kinase domain of EGFR, alters the binding of erlotinib to the ATP-binding pocket [23–26]. ERBB3 signaling activation, which bypasses the EGFR signaling pathway, was found in the EGFR-TKI resistant cells derived from HCC827 cells [20]. Recently, reactivation of ERK1/2 was shown to occur after continuous exposure to EGFR-TKI for 72 hours, and led to resistance to EGFR-TKI [16]. Our results in HCC827 cells show that after 72 hours of erlotinib treatment, YAP protein expression rebounds and ERBB3 mRNA expression increases. One explanation for these results is that upregulation of YAP increases ERBB3 expression, and the ERBB3 signaling pathway bypass EGFR signaling pathway to reactivate ERK1/2.

In our study, merlin (NF2), LATS1 protein expression and p-YAP/YAP ratio decreased in HCC827 ER cells. In these cells, decreasing NF2 and LATS1 expression may have led to increasing YAP stability in HCC827 ER cells. Furthermore, YAP and ERBB3 protein expression, and mRNA expression also increased in HCC827 ER cells. These findings suggest that increasing YAP expression activates ERBB3 expression in erlotinib-resistant HCC827 cells. In addition, down-regulation of NF2 may correlate with increasing YAP protein expression which in turn activates ERBB3 expression (Supplementary Figure S3). Our hypothetical model is that YAP may promote erlotinib resistance through an autocrine loop with the ERBB3 pathway (summarized in Figure 6G and Supplementary Figure S3).

T790M mutation renders erlotinib unable to block EGFR signaling, and leads to the resistance to erlotinib [23–26]. We found that inhibition of YAP by siRNA increase erlotinib's cytotoxicity to H1975 cells. Verteporfin is a small molecule that inhibits TEAD-YAP interaction, and several studies have used it as YAP inhibitor to suppress YAP-induced tumorigenesis [10, 14, 27–29]. In our study, we verified that verteporfin decreased YAP protein expression, GTIIC reporter activity and mRNA expression of downstream genes AREG and CTGF in H1975 cells. Our results suggest that verteporfin has an effect similar to that of YAP siRNA on inhibiting YAP, and less likely through off-target effects [30]. Verteporfin increased sensitivity of H1975 cells to erlotinib and in combination with erlotinib, synergistically reduced migration, invasion and tumor sphere formation abilities in H1975 cells. Previous studies also reported that inhibition of YAP increased sensitivity of ovarian cancer and NSCLC to erlotinib [27,31]. However, these studies, unlike ours, did not show that direct YAP forced overexpression by YAP plasmid transfection in erlotinib-sensitive NSCLC cell line HCC827 promoted erlotinib resistance. Moreover, our manuscript provides key experiments showing that forced YAP overexpression by YAP plasmid transfection in HCC827 cells increases ERBB3 expression and decreases the sensitivity to erlotinib which was not shown in these studies [27,31].

In conclusion, our study indicates that YAP forced overexpression promotes erlotinib resistance in human NSCLC, and inhibition of YAP increases cytotoxicity of erlotinib in erlotinib resistant-NSCLC. The development of drugs that target YAP for erlotinib-resistant human NSCLC may be warranted.

## MATERIALS AND METHODS

### Cell culture and generation of HCC827 ER

Human NSCLC cell lines H1975 and HCC827 were obtained from American Type Culture Collections (Manassas, VA). Cell lines were maintained in RPMI-1640 supplemented with 10% heat-inactivated fetal bovine serum (FBS), penicillin (100 mg/ml) and streptomycin (100 mg/ml), and were cultured at 37°C in a humid incubator with 5% CO_2_.

The HCC827 (erlotinib-resistant) ER cell line was established by culturing HCC827 cells in 5% FBS culture media containing erlotinib. Cells were maintained at the initial erlotinib concentration of 0.1 μM (IC50). The dose of erlotinib was titrated gradually to the final concentration of 10 μM after 12 weeks. Cells were diluted and subculture to a 96-well plate, and only single-cell cloning with dividing ability was chosen. HCC827 ER cells were established and then were maintained in RPMI-1640 medium with 10% FBS containing 10 μM erlotinib.

### Small molecule treatment, SiRNA, and plasmid DNA transfection

The EGFR-TKI erlotinib was purchased from Selleckchem (Houston, TX). The YAP/TEAD inhibitor verteporfin was purchased from Sigma Aldrich (St. Louis, MO). The SMARTPool siRNA targeting YAP was purchased from Thermo Scientific Dharmacon (Pittsburgh, PA), and merlin siRNA was purchased from cell signaling (Cell Signaling Technology, Inc). The YAP plasmid DNA used to over-express the YAP gene in the cells was purchased from Addgene (Cambridge, MA). Cells were plated in 6-well plates (for western blot) and 24-well plates (for PCR and reporter assay) for 24 hours before treatment. Small molecule inhibitors, erlotinib and verteporfin were dissolved in DMSO. Cells were treated with erlotinib and verteporfin at different dosages, and were grown for 24 hours before being harvested. For time-dependent treatment, cells were harvested at 0, 8, 16, 24, 48, 72 hours after treatment with erlotinib alone and with erlotinib and verteporfin combined. Cells were transfected with 4μg of YAP plasmid DNA using Lipofectamine 2000 transfection reagent, and 100 nmol/L of siRNA using Lipofectamine RNAiMAX (Invitrogen, Carlsbad, CA) according to the manufacturer's protocol. After transfection for 48 hours, cells were harvested for further analysis.

### RNA isolation, cDNA synthesis, and quantitative real-time RT-PCR

The RNeasy Mini kit (Qiagen, Valencia, CA) was used for total RNA extraction from cells. Total RNA was then transcribed to the cDNA by using iScript cDNA Synthesis Kits (Bio-Rad, Hercules, CA), according to the manufacturer's protocol. The amount of total RNA used for cDNA transcription was 500 ng, and the cDNA was used as the template for real-time PCR. TaqMan Technology on an Applied Biosystems 7000 sequence detection system (Applied Biosystems, Foster City, CA) was used for real-time PCR detection. ERBB3, AREG and CTGF gene expression and endogenous control gene b-glucuronidase (GUSB) were detected by using primers and probe sequences commercially available (Applied Biosystems) and analyzed using Relative Quantification Software (Applied Biosystems).

### Luciferase reporter assay

The cell lines were transfected with 8 × GTIIC-luciferase plasmid (Addgene, Cambridge, MA) and Renilla luciferase pRL-TK plasmid (Promega, Madison, WI). The transfection reagent for SiRNA treatment was Lipofectamine RNAiMAX, and Lipofectamine 2000 (Invitrogen, Carlsbad, CA) for small molecule inhibitors. After 48-hour transfection and treatment, cells were harvested and transferred into a 96-well plate. Dual-Luciferase Reporter Assay Kit (Promega, Madison, WI) was used for analysis, and luminescent signaling was detected by using a GloMax-96 Microplate Luminometer (Promega, Madison, WI) according to the manufacturer's instructions.

### Western blot analysis

For protein extraction, the cell lines were lysed by using M-PER Mammalian Protein Extraction Reagent (Thermo) supplied with Complete Protease Inhibitor Cocktails (Roche, Lewes, UK). The total amount of protein for each sample was 20μg, and the samples were run on 4~20% gradient SDS–polyacrylamide gels (Bio-Rad Laboratories, Inc., Hercules, CA) and then were transferred to Immobilon-Pnitrocellulose membranes (Millipore, Bellerica, MA). The membranes were probed with primary antibodies YAP, p-YAP(S127), merlin, LATS1, TAZ, ERBB3, p-ERBB3 (Cell Signaling Technology, Inc) and GAPDH (Sigma-Aldrich) in 4°C overnight after being blocked with 5% non-fat milk. The membranes were then incubated with appropriate second antibodies, as well as anti-rabbit bodies for YAP, p-YAP(S127), merlin, LATS1, TAZ, ERBB3 and p-ERBB3 and anti-mouse antibody for GAPDH at room temperature for 1 hour, and finally were detected by using an ECL blotting analysis system (Amersham Pharmacia Biotech, Piscataway, NJ)

### Cell viability assay

Cells were cultured in a 96-well plate and treated with different doses of erlotinib and verteporfin (0, 0.01, 0.03, 0.1, 0.3, 1, 3, 10, 30, 100 μM). For the combination treatment, we combined a constant dose of 1.0 μM verteporfin with different doses of erlotinib (0, 0.01, 0.03, 0.1, 0.3, 1, 3, 10, 30, 100 μM). After 72 hours of incubation, cells were lysed and CellTiter-Glo Luminescent Cell Viability Assay reagent (Promega) was added to generate luminescent signaling. Luminescent signaling was detected by using the GloMax-96 Microplate Luminometer. Proportional cell viability was analyzed with GraphPad Prism 5.0 software (GraphPad Software, Inc., La Jolla, CA), which was used to calculate dose-response curves and IC50.

### Wound-healing assay

H1975 cells were sub-cultured in 6-well plates to the condition of confluence. The plates were scratched by a 200 μl pipette tip, and then the cells were treated with DMSO, erlotinib alone, verteporfin alone and the combination of erlotinib with verteporfin. The cells were grown continuously, and phase contrast images were taken at the time of the scratch (0 h) and 18 hours.

### Transwell invasion assay

A six-well plate transwell system (Corning Incorporated, USA) was used for transwell invasion assay, and the transwell inserts were coated with 100 μl matrigel and incubated at 37°C for 2 hours. H1975 cells were tripsinized and resuspended in serumfree medium, and the cells were seeded on the upper chamber of the transwell. Erlotinib and verteporfin were added into the upper chamber of the transwell, and the lower chamber was infused with 2 ml complete growth medium (10% FBS). The gel and cells in the upper chamber of transwell were wiped after incubation at 37°C for 20 hours. The membrane was stained by hematoxylin for 40 seconds after methanol fixation. Finally, phase contrast images were taken with a Primo Vert microscope (ZEISS, Gottingen, Germany) and the cells on the lower side of the membrane were counted.

### Sphere formation assay

H1975 cells were tripsinized and resuspended in serumfree medium, and single-cell suspensions were plated (5000 cells/well) in 24-well ultra-low attachment plates (Corning) and cultured with stemPro mesenchymal stem cells in serum-free culture medium (MSC-SFM) with L-glutamin and penicillin (100 mg/ml) and streptomycin (100 mg/ml) supplement. The cells were treated with DMSO, erlotinib alone, verteporfin alone and the combination of erlotinib with verteporfin, and continuously cultured at 37°C in a humid incubator with 5% CO2 for 72 hours. Phase contrast images were taken and the number of spheres was counted after treatment.

### Statistical analysis

Data are shown as mean ± standard deviation (SD) from three independent experiments. GraphPad Prism (Version 5.0; GraphPad Software, San Diego, CA, USA) was used for all statistical analyses. Student's t-test was used for comparison between two groups. One-way ANOVA followed by Turkey multiple comparisons were used to compare differences among multiple groups. All P values were 2-sided and *P*<0.05 was considered statistically significant.

## SUPPLEMENTARY FIGURES


