# Supplementary material for: YAP promotes erlotinib resistance in human non-small cell lung cancer cells
Source: Oncotarget. 2016 Jul 7;7(32):51922–33. doi: 10.18632/oncotarget.10458 (PMC5239524; doi:10.18632/oncotarget.10458)
Supplement: Supplementary file 1 [file oncotarget-07-51922-s001.pdf]

YAP promotes erlotinib resistance in human non-small cell lung cancer cells

SUPPLEMENTARY FIGURES

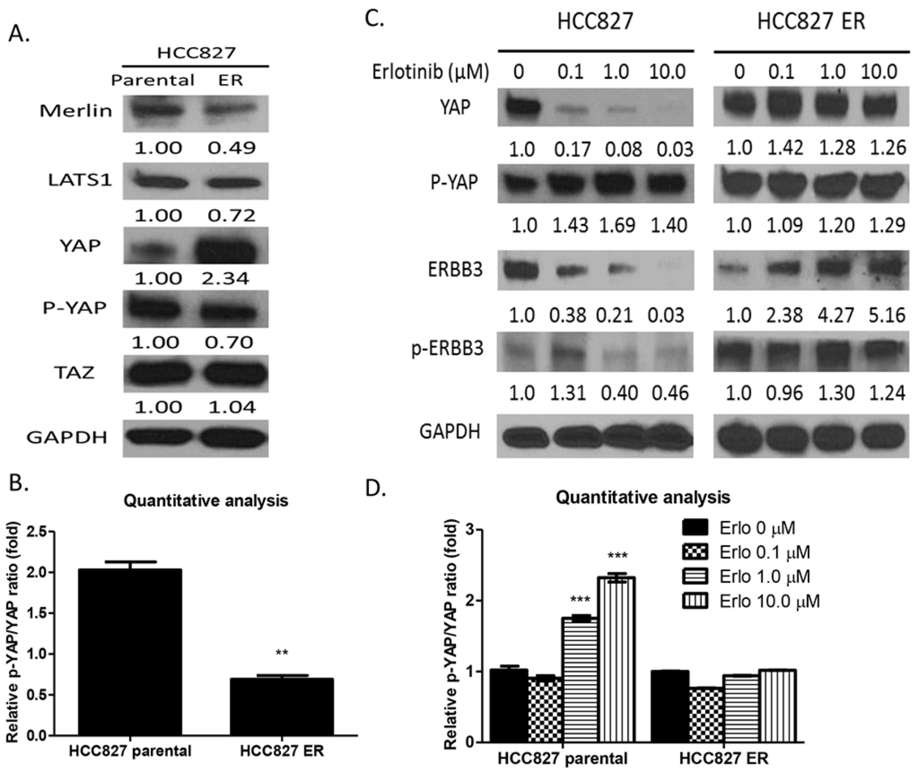

Supplementary Figure S1: Western blot analysis of merlin, LATS1, YAP, p-YAP, TAZ, ERBB3 and p-ERBB3 protein expression in parental HCC827 and HCC827 ER cells. A. Merlin and LATS1 protein expression decreased in HCC827 ER cells, and YAP protein expression increased in HCC827 ER cells. TAZ protein expression level did not change between parental HCC827 and HCC827 ER cells. **B.** Quantitative analysis of the p-YAP/YAP protein ratio between parental HCC827 and HCC827 ER cells. The ratio significantly decreased in HCC827 ER cells as compared to parental HCC827 cells (**P < 0.01). **C.** YAP, p-YAP, ERBB3, and p-ERBB3 protein expression in parental HCC827 and HCC827 ER cells after various doses of erlotinib . YAP, ERBB3 and p-ERBB3 protein expression decreased in a dose-dependent manner in parental HCC827 cells, and ERBB3 protein expression increased in HCC827 cells after higher dose erlotinib treatment. **D.** Quantitative analysis of the p-YAP/YAP protein ratio between parental HCC827 and HCC827 ER cells after various doses of erlotinib. The ratio significantly decreased after 1.0μM and 10.0μM doses of erlotinib in parental HCC827 cells (***P < 0.001).

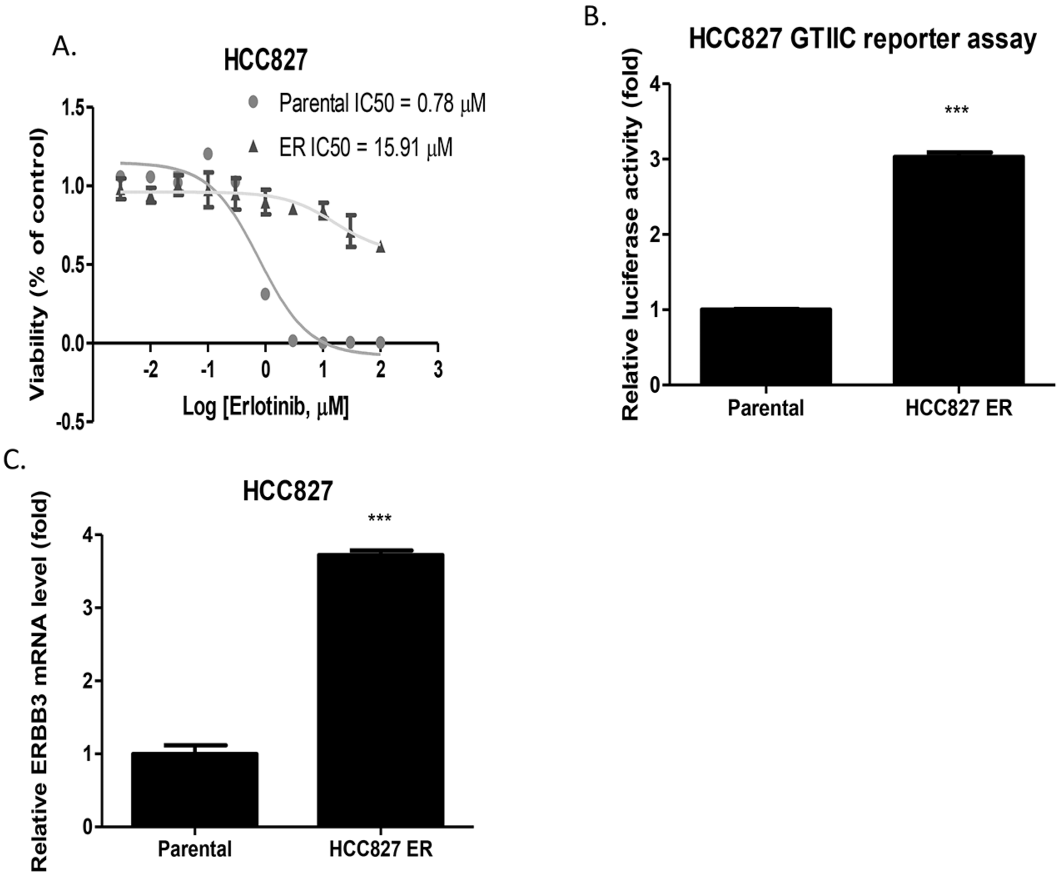

Supplementary Figure S2: Cell viability analysis in HCC827 ER and parental HCC827 cells after erlotinib treatment, and GTIIC reporter activity, and ERBB3 mRNA expression between parental HCC827 and HCC827 ER cells. **A.** The IC50 of erlotinib was 15.91 μM for HCC827 ER cells, and 0.78 μM for parental HCC827 cells. **B.** GTIIC reporter activity was significantly higher in HCC827 ER cells than in parental HCC827 cells. **C.** ERBB3 mRNA expression was also significantly higher in HCC827 ER cells than in parental HCC827 cells (*** $P < 0.001$).

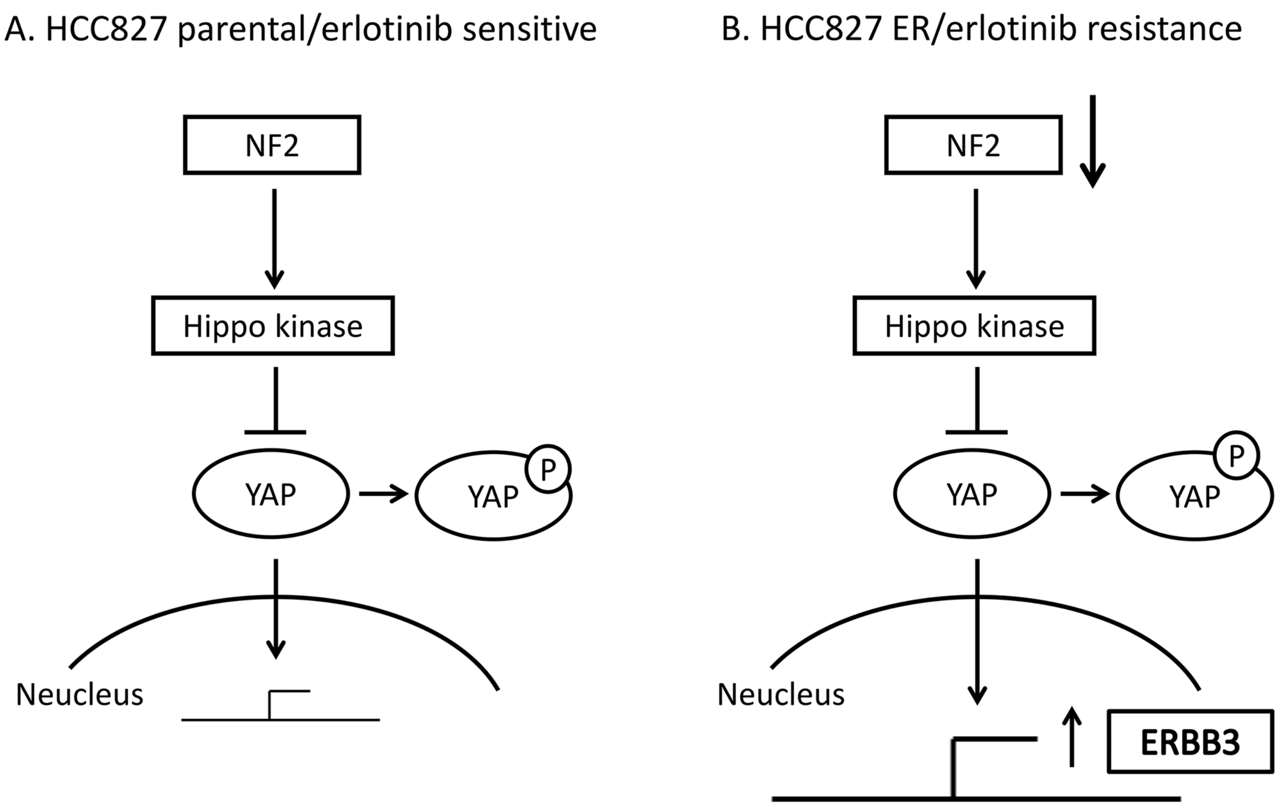

Supplementary Figure S3: Our hypothetical model showing that in HCC87 ER cells, a decrease in merlin (NF2) leads to increased YAP expression and then activates ERBB3 expression.
